# High throughput proteomic analysis of the secretome in an explant model of articular cartilage inflammation

**DOI:** 10.1016/j.jprot.2011.02.017

**Published:** 2011-05-01

**Authors:** Abigail L. Clutterbuck, Julia R. Smith, David Allaway, Pat Harris, Susan Liddell, Ali Mobasheri

**Affiliations:** a Musculoskeletal Research Group, Division of Veterinary Medicine, School of Veterinary Medicine and Science, Faculty of Medicine and Health Sciences, University of Nottingham, Sutton Bonington Campus, Leicestershire, LE12 5RD, United Kingdom; b Bruker UK Limited, Coventry, CV4 9GH, United Kingdom; c WALTHAM Centre for Pet Nutrition, Waltham-on-the-Wolds, Melton Mowbray, Leicestershire, LE14 4RT, United Kingdom; d Proteomics Laboratory, School of Biosciences, Faculty of Science, University of Nottingham, Sutton Bonington Campus, Leicestershire, LE12 5RD, United Kingdom

**Keywords:** Articular cartilage, Osteoarthritis (OA), Explant culture, High-throughput proteomics, Mass spectrometry, Secretome

## Abstract

This study employed a targeted high-throughput proteomic approach to identify the major proteins present in the secretome of articular cartilage. Explants from equine metacarpophalangeal joints were incubated alone or with interleukin-1beta (IL-1β, 10 ng/ml), with or without carprofen, a non-steroidal anti-inflammatory drug, for six days. After tryptic digestion of culture medium supernatants, resulting peptides were separated by HPLC and detected in a Bruker amaZon ion trap instrument. The five most abundant peptides in each MS scan were fragmented and the fragmentation patterns compared to mammalian entries in the Swiss-Prot database, using the Mascot search engine. Tryptic peptides originating from aggrecan core protein, cartilage oligomeric matrix protein (COMP), fibronectin, fibromodulin, thrombospondin-1 (TSP-1), clusterin (CLU), cartilage intermediate layer protein-1 (CILP-1), chondroadherin (CHAD) and matrix metalloproteinases MMP-1 and MMP-3 were detected. Quantitative western blotting confirmed the presence of CILP-1, CLU, MMP-1, MMP-3 and TSP-1. Treatment with IL-1β increased MMP-1, MMP-3 and TSP-1 and decreased the CLU precursor but did not affect CILP-1 and CLU levels. Many of the proteins identified have well-established extracellular matrix functions and are involved in early repair/stress responses in cartilage. This high throughput approach may be used to study the changes that occur in the early stages of osteoarthritis.

## Graphical Abstract

**Figure:**
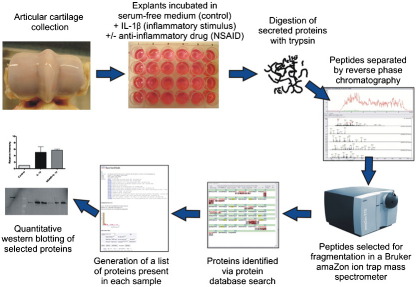

**Research Highlights:**

► High-throughput proteomics identified major cartilage secretome proteins. ► Western blotting confirmed the presence of CILP-1, CLU, MMP-1, MMP-3 and TSP-1. ► Treatment with IL-1β increased MMP-1, MMP-3 and TSP-1. ► The proteins identified are involved in cartilage function and early stress responses. ► This approach may be used to study changes in the early stages of osteoarthritis.

## 1 Introduction

Articular cartilage is a mechanically resilient connective tissue responsible for load-bearing and low-friction movement in the synovial joints of all vertebrates [1]. The extracellular matrix (ECM) of cartilage gives the tissue resilience and elasticity. The ECM consists of three classes of molecules: collagens, aggregating proteoglycans, and noncollagenous proteins. Type II, IX, and XI collagens form a fibrillar framework of macromolecules that give the tissue form, tensile stiffness and mechanical strength [2,3]. Large aggregating proteoglycans (predominantly aggrecan) allow cartilage to swell and resist compressive forces [4,5]. Small proteoglycans including decorin, biglycan, and fibromodulin, bind to other matrix macromolecules and help to stabilise the ECM. Other collagenous and non-collagenous macromolecules present within the ECM perform a variety of structural and informational roles, facilitate cell–cell and cell–matrix interactions and bind growth factors [5,6]. The chondrocyte is the only cell type present in articular cartilage [7]. During embryonic development chondrocytes synthesise a cartilaginous template for endochondral ossification and skeletal development and in postnatal life they maintain the ECM by regulating the turnover of matrix components in response to biomechanical, biochemical and endocrine signals [8]. Chondrocytes actively synthesise new ECM components as well as the proteolytic enzymes responsible for catabolic breakdown of cartilage. The matrix metalloproteinases (MMPs), ADAMs (A Disintegrin And Metalloproteinase) and ADAMTSs (A Disintegrin And Metalloproteinase with Thrombospondin Motifs) are the main proteolytic enzymes that regulate ECM turnover in cartilage [9].

Osteoarthritis (OA) is a degenerative disease of synovial joints that involves joint inflammation, bone remodelling and destruction of the articular cartilage component [10,11]. In OA there is an imbalance between the synthesis and degradation of ECM macromolecules [12]. This can be due to increased enzymatic activity of MMPs [13], and pro-inflammatory mediators such as cytokines [14], prostaglandins and nitric oxide [15], coupled with the reduced anabolic capacity of chondrocytes [16] and the tissue's inherently poor reparative capacity due to its avascular nature [7]. Consequently OA is characterised by the loss of structural constituents from the ECM. The degradation and release of proteins and glycoproteins from cartilage in OA can vary according to the stage of the disease process. For example, elevated serum cartilage oligomeric matrix protein (COMP) is correlated with the presence of OA and disease severity [17]. Therefore, the ability to detect biomarkers of cartilage degradation and/or inflammation in biological samples, such as serum, urine or synovial fluid, may enable clinicians to diagnose sub-clinical OA as well as determining the disease stage in both human and companion animals. Identifying these biomarkers will also aid drug discovery and drug safety/efficacy monitoring in patients and in animal models. Using combinations of biomarkers may be more effective in achieving these goals, thus having a panel of biomarkers will help researchers and the pharmaceutical industry to monitor disease progression as well as to assess responses to treatment in experimental models of OA [18,19].

Proteomics is being increasingly applied in basic cartilage biology [20] and OA research [21]. Characterisation of cell lysates from isolated chondrocytes has yielded valuable information regarding the intracellular proteins of the chondrocyte proteome, and paved the way for future studies on cartilage pathologies such as OA [22,23]. Studies of soluble proteins in cartilage tissue from OA patients have increased the knowledge of the proteins contained within the ECM of diseased versus normal tissue [24]. A number of papers have reported on proteins secreted from the cartilage ECM in response to pathological insults such as interleukin (IL)-1α and all-trans-retinoic acid [23,25,26], IL-1β and [27] and mechanical compression [28–30]. Identifying proteins released from cartilage explants has the potential to give an indication of disease biomarkers likely to be present in the synovial fluid or blood of patients in the early stages of OA. The cartilage explant model also maintains and supports chondrocytes within their natural physicochemical microenvironment. This *in vitro* model is popular because it can overcome many of the complicating variables associated with the de-differentiation of chondrocytes in monolayer culture as well as the challenges of interpreting data from animal models and *in vivo* work. The horse is a suitable species from which cartilage is used to model human OA, owing to the fact that horses are athletic animals that can suffer from joint injuries similar to those in human athletes. The equine cartilage explant model is well established in our laboratory and has been used to develop *in vitro* cultures mimicking joint inflammation using pro-inflammatory cytokines. For these reasons, the present study used healthy equine cartilage explants cultured in serum-free media, either alone (representing healthy cartilage), in the presence of the pro-inflammatory cytokine equine IL-1β (equine recombinant protein) to replicate the early inflammatory stages of OA, or a combination of IL-1β and carprofen (a COX-2 specific non-steroidal anti-inflammatory drug (NSAID) commonly used to treat arthritic symptoms and joint inflammation) to simulate anti-inflammatory pharmacotherapy. Our aim was to use proteomic analysis of the explant culture media to identify the major proteins secreted from cartilage.

## 2 Materials and methods

### 2.1 Cartilage explant culture

Macroscopically normal articular cartilage was obtained from the weight bearing regions of the metacarpophalangeal joints of three horses. The animals used in this study were sourced from the abattoir and were euthanized for purposes other than research. The study received the full approval of the local ethics committee but since abattoir tissues were used it was exempt from review by animal welfare authorities. Articular cartilage from individual animals was kept separate throughout the study. Cartilage shavings of equal thickness were aseptically harvested into low glucose (1 g/L glucose) Dulbecco's Modified Eagle's Medium (DMEM) (HyClone) containing 4% penicillin/streptomycin (Gibco) before being washed twice in PBS for 20 min. Cartilage shavings from each animal were cut into 3 mm discs using a sterile biopsy punch and five discs/well were placed into 18 wells, containing 1 ml of DMEM supplemented with 2% penicillin/streptomycin. Plates were incubated overnight (37 °C/5% CO_2_). Media was then replaced with fresh media.

### 2.2 Experimental design

**Fig. 1:**
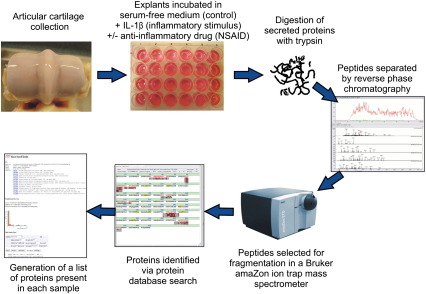
Schematic overview of the experimental design used in this study.

Explants from three separate animals were subjected to three different treatments; control, IL-1β and NSAID + IL-1β. Six replicates per treatment for each of the 3 animals were used totalling to 18 samples per animal, and altogether equalling to 54 samples in total. All wells contained 1 ml of the culture medium. Control wells contained the culture media alone. Recombinant equine IL-1β (10 ng/ml; R&D Systems) was added to the remaining wells to induce cartilage inflammation. IL-1β alone formed the negative control and the addition of a NSAID, carprofen (100 μg/ml; Rimadyl®, Pfizer Animal Health), to the remaining IL-1β-treated wells acted as a positive control to counteract the IL-1β-stimulated inflammation. Explants were incubated at 37 °C and 5% CO_2_ for six days. The viability of the chondrocytes in explants treated for six days with IL-1β- was checked using a LIVE/DEAD® Cell Viability Assay (Invitrogen). Cell viability was unaffected by exposure to IL-1β (10 ng/ml) for this period of time (data not shown). After six days, the supernatants were removed and split into two aliquots before freezing at − 20 °C. A schematic overview of the experimental design is shown in Fig. 1.

### 2.3 Trypsin digestion of soluble proteins

Protein content of the samples was quantified using a Detergent Compatible (DC) Protein Assay (Bio-Rad). Dithiothreitol (DTT, Sigma-Aldrich) was added to 100 μl of each sample to a final concentration of 10 mM and incubated at 37 °C for 30 min. Iodoacetamide (Sigma-Aldrich) (55 mM) was added to each sample and incubated for 45 min at 37 °C in the dark. Ice-cold acetone (Sigma-Aldrich) was added to each tube and mixed well. Samples were incubated on ice for 1 h, vortexing for 10 s every 15 min. The precipitate was then centrifuged at 15,000 *× g* for 5 min at 4 °C and the supernatant was discarded. The pellet was air dried and re-suspended in 50 mM ammonium bicarbonate (Sigma-Aldrich) solution to a final concentration of 10 μg/mL. Mass Spectrometry Grade trypsin (Promega) was added to each tube at a ratio of 1:50 enzyme/target protein and incubated overnight at 37 °C. The reaction was stopped by adding 1 μl of formic acid (Sigma-Aldrich). Trypsin digested samples were stored at − 80 °C until analysis by ESI mass spectrometry.

### 2.4 Analysis by LC–MS/MS

Liquid chromatography was performed using an Easy-nLC (Bruker UK Ltd) under the control of Hystar (Bruker UK Ltd). Peptides generated by tryptic digestion were prepared for LC–MS/MS as follows; 2 μl of digest was added to 48 μl of solvent A (95%(v/v) H_2_O, 5%(v/v) acetonitrile (Sigma-Aldrich), and 0.1%(v/v) formic acid (Sigma-Aldrich)). A volume (5 μl) of this dilution was then loaded onto a C18 Pepmap column (75 μm ID, 15 cm, LC Packings). Peptides were separated at a flow rate of 300 nl/min and the introduction of solvent B (95%(v/v) acetonitrile, 5%(v/v) water, and 0.1%(v/v) formic acid) over a 30 min linear gradient (solvent B went from 0% to 5% in 30 s, then from 5% to 55% in 22 min, up to 95% in 4 min, was held at 95% for 2 min 30 s to wash the column, then returned to 5% over 1 min followed by 10 min of equilibration in 5% before the next injection, total run time 40 min). The LC system was interfaced directly with a 3-D high capacity ion trap mass spectrometer (amaZon, Bruker Daltonics) via the nanoESI spray source and a target of 200,000 was set for SmartICC™.

Up to five precursor ions above a threshold of 10,000 were selected per MS scan. Each precursor was fragmented twice and then the mass was excluded for 1 min. Singly charged ions were excluded. SPS tuning was allowed for transmission of the precursor into the trap. SmartFrag™ controlled the fragmentation of each precursor ion and this was achieved using helium gas and a 30–200% collision energy range with an amplitude of 1.3 V.

### 2.5 Data processing and analysis

Raw LC–MS/MS data were processed automatically and Mascot compatible files (*.mgf) created using DataAnalysis^TM^ 4.0 software (Bruker UK Ltd) with the following parameters: compounds (autoMS) threshold 10,000, number of compounds was unlimited, retention time windows were 1 min for C18 (30 min gradient).

Database searches were performed using Mascot [31] software and the Swiss-Prot database (version 51.6), with the following parameters: 2+ and 3+ ions, peptide tolerance 0.5 Da, ^13^C = 1, fragment tolerance 0.5 Da, missed cleavages: 1, and instrument type: ESI-TRAP. Fixed modifications: carbamidomethyl cysteine and variable modifications: oxidation of methionine, deamidation of asparagine and glutamine residues. Individual MS/MS spectra for peptides with a Mascot Mowse score^1^ lower than 40 were inspected manually and only included in the statistics if a series of at least four continuous fragment ions were observed.

### 2.6 Submission of MS data to PRIDE

Fragmentation spectra, protein and peptide identifications from further Mascot searches of the most recent SwissProt database (UniProtKB Release 2011_01) are available in the PRIDE database [32] (http://www.ebi.ac.uk/pride/) at the European Bioinformatics Institute under accession number 15852. The data was converted using PRIDE Converter [33] (http://code.google.com/p/pride-converter).

### 2.7 Western blot validation of selected proteins

Western blotting was used to confirm the presence of a representative group of proteins identified by high throughput proteomics. Replicate samples for each treatment were combined to give a total of three samples per animal. The protein content of each sample was quantified using a DC Protein Assay (Bio-Rad) and concentrated by freeze-drying (Heraeus-Christ) to yield 50 μg of total protein per sample. Proteins extracted from equine cartilage shavings were used as positive controls. The tissue was frozen in liquid nitrogen and crushed into a fine powder in a pre-cooled mortar and pestle. Supernatant and cartilage control samples were re-suspended in NuPAGE® LDS sample buffer (Invitrogen), and loaded onto 4–12% NuPAGE® buffered gradient pre-cast Bis/Tris gels (50 μg protein/lane, Invitrogen). Samples were electrophoresed for 1 h under reducing conditions at 200 V. Gels were electro-transferred onto polyvinyl difluoride (PVDF, Invitrogen) membranes for another hour at 30 V. After blocking in Marvel/Tween (5% nonfat dry milk powder, 0.05% Tween 20 in TBS), primary antibodies were diluted as recommended by the manufacturer in Marvel/Tween and added to the membrane for overnight incubation at 4 °C.

The specificity and cross-reactivity of each of the commercial antibodies used in this study was validated by analysis of the total proteins extracted from equine cartilage shavings. CILP-1 blots were probed using a polyclonal sheep antibody (1:300 dilution, R&D Systems). Clusterin (1:500 dilution, Santa Cruz) and MMP-3 (1:1000 dilution, Abcam) blots were probed with goat polyclonal antibodies. A rabbit polyclonal to MMP-1 (Abcam) was used at a dilution of 1:5000. TSP-1 (1:50 dilution, Santa Cruz) and TSP (1:1000 dilution, Abcam) blots were probed with mouse monoclonal antibodies. All the antibodies selected for use in this study were produced against human peptide sequences with 99–100% identity to their counterparts in *Equus caballus*. The peptide sequences used by each manufacturer for antibody production were checked using the NCBI Basic Local Alignment Search Tool (Protein BLAST; http://blast.ncbi.nlm.nih.gov/Blast.cgi?PAGE=Proteins).

Appropriate secondary antibodies conjugated to horseradish peroxidise (HRP) were used diluted in Marvel/Tween; polyclonal sheep HRP (diluted 1:10,000, Abcam), polyclonal rabbit anti-goat immunoglobulins/HRP (diluted 1:10,000, Dako) and anti-mouse/anti-rabbit labelled polymer HRP (diluted 1:400, Dako).

Chemiluminescence was used to detect the protein bands using ECL + (GE Healthcare) on a Typhoon Trio+ Variable Mode Imager (GE Healthcare). Densitometric quantification of the western blot bands was performed using ImageJ software (http://rsbweb.nih.gov/ij/; National Institutes of Health, Bethesda, MD). The relative intensity of protein bands in IL-1β and NSAID + IL-1β treated samples was compared to those in control samples from each animal.

## 3 Results

**Fig. 2:**
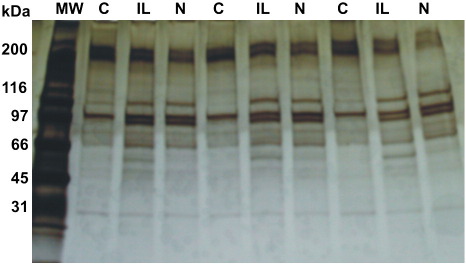
Silver-stained 1-dimensional gel (1-D) protein profiles of medium from cartilage explants incubated alone (control, C), or treated with equine interleukin-1beta (β) (IL), or with a non-steroidal anti-inflammatory drug plus IL combined (N). Each set of 3 lanes labelled C, IL and N represent samples from a different animal.

Silver staining of 1-D gels revealed differences in the secretome protein profiles between control, IL-1β-treated and IL-1β plus NSAID-treated samples (see Fig. 2). The differences observed by 1-D PAGE and silver staining suggested that there are alterations in the secretomes of differently treated cartilage explants and catalysed the subsequent phases of this work.

The majority of the secreted proteins identified using LC–MS/MS with high Mowse scores in multiple samples were cartilage ECM proteins or proteins with well-established matrix functions (see Table 1). Abundant proteins that were identified in the majority of the samples from all animals included aggrecan core protein, fibronectin, decorin and COMP. The database search identified some proteins more frequently (in terms of Mascot hits) in the IL-1β-treated samples compared to the untreated controls, namely MMP-3 and TSP-1.

Cross species peptide matching [34,35] enabled the identification of proteins for which the equine versions were absent or incomplete. Some peptides, such as decorin, matched proteins from multiple species (human, horse, cow, rat, dog and rabbit). Where the horse sequence was present in the database, it generally achieved the top Mascot Mowse score (e.g. 156 compared in the case of decorin) compared to the closest homologue. The only exception was fibronectin, which achieved a top Mascot Mowse score of 642 with the bovine sequence but only 221 with the equine sequence. This is likely to be due to the equine entry in the database being considerably truncated. Where the horse sequence was absent from the database, multiple species matching increased confidence in the protein detected, for example, aggrecan core protein which had peptide matches with human, mouse, rat, pig and dog. Some peptides only matched proteins from one species such as equine MMP-3, or two species such as equine and bovine MMP-1.

The majority of the proteins identified in this study were ECM components or ECM-associated, however, a small number of non-structural proteins were also found. Ten non-ECM proteins were present in multiple samples and are shown in Table 2. Many of these had low Mowse scores under 55, except serum albumin precursor with a score of 71.

Many proteins were only identified in a single sample from the 54 samples tested (18 samples per treatment). Table 3 details peptides from these proteins that contained four or more continuous fragment ions in a series. The presence of four or more fragment ions in a row was defined as the criterion for which a single peptide match was considered to be a true positive, rather than five fragment ions as is standard. Some of the peptides were small and only consisted of five or six amino acids and the detection of all the fragment ions is unlikely, therefore the detection of four ions in a row was set as the confirmation limit. This enabled the identification of four extra proteins; glucose-6-phosphate 1-dehydrogenase 2, centromere protein C1, lactadherin and max-like protein X, as shown in Table 3. The presence of some of the proteins in Table 3 may be due to contamination, for example keratin from hair, skin or nails during tissue collection, culture or sample processing [36]. Table 4 shows the proteins found in only one sample, which have peptides with fewer than four continuous fragment ions in a series. These proteins have low Mowse scores of 55 or less.

**Fig. 3:**
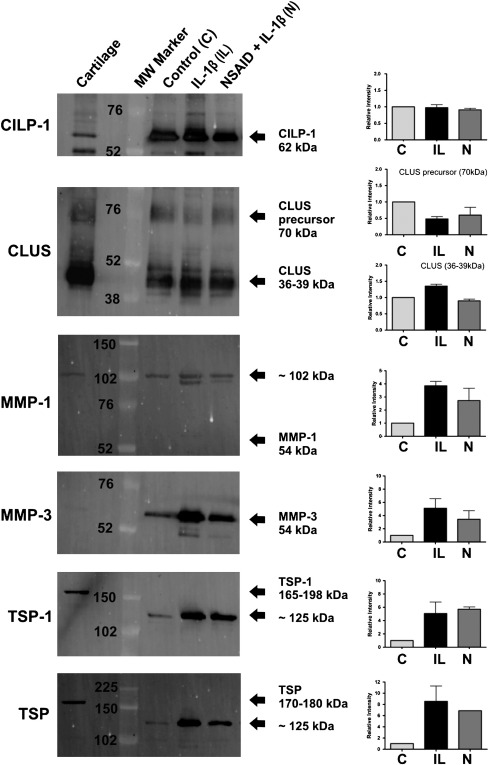
Western blot images of explant medium from cartilage explants alone (control, C), and treated with equine interleukin-1beta (β) (IL), or with IL-1β and a non-steroidal anti-inflammatory drug (N) combined. Blotting for cartilage intermediate layer protein-1 (CILP-1), clusterin (CLU), matrix metalloproteinase (MMP)-1 and MMP-3, thrombospondin (TSP)-1 and a pan antibody to 4 forms of TSP. Arrows indicate observed molecular weights (MW) in kilo Daltons (kDa).

The tables presented do not provide any quantitative information about differences among control, IL-1β-treated and IL-1β plus NSAID-treated samples with regard to the abundance of the proteins listed. The approach used only determined the presence or absence of proteins. Therefore quantitative western blotting was used to validate and quantify a selected number of the proteins identified. This approach confirmed the presence of selected matrix associated proteins and helped quantify the relative intensities of the protein bands in the IL-1β-treated and IL-1β plus NSAID-treated samples versus the untreated controls. Western blotting confirmed the presence of CILP-1, CLU, MMP-1, MMP-3 and TSP-1 in the samples (Fig. 3). IL-1β-treated samples showed increased protein levels of MMP-1, MMP-3 and TSP-1 in comparison to controls and MMP-3 was actually present in lower amounts in the NSAID-treated samples. The weak band(s) in the tissue homogenate controls showed that MMP-3 was present in cartilage, albeit at much lower concentrations.

## 4 Discussion

Proteomics is increasingly applied in basic and clinical rheumatology and orthopaedics to help understand the biology of joint tissues, responses to pro-inflammatory cytokines and growth factors as well as mechanical signals [23,27,29,30]. Proteomic techniques also have the potential to identify novel biomarkers of joint diseases. In this study we set out to refine a serum-free explant model of articular cartilage inflammation and tested the feasibility of using it for studying the major proteins present in the secretome. The explant model was originally established in our laboratory with the intention of using it as a screening system for testing the effects of putative anti-inflammatory compounds from plant extracts [37] as well as identifying biomarkers of early OA. High throughput proteomics and cross species peptide matching were then used to identify the main proteins present in the secretome. This approach has a key advantage: it does not require gel electrophoresis and is more rapid than traditional 2-D gel MALDI-TOF MS analysis.

Using comparative proteomic analysis, we identified several constitutively expressed ECM molecules in the supernatants of cartilage explants, namely aggrecan, fibromodulin, decorin, biglycan and COMP. These proteins have been identified in proteomic studies in human [24] and mouse [25] cartilage, thus facilitating the validation of our equine cartilage model. One of the most abundant proteins in the explant media was COMP, a non-collagenous matrix protein that mediates chondrocyte attachment to the ECM [38] and organises ECM assembly [39]. COMP has been suggested as a potential biomarker for OA and it has been detected in synovial fluid, serum and urine from human and equine OA patients [17,40–44]. Moreover, its presence correlates with disease severity and progression. The presence of COMP in this model, concurs with that of other *in vitro* studies that have found COMP abundant in media exposed to cartilage explants [24,25,28].

Western blotting confirmed that IL-1β-treatment increases the abundance of MMP-3 in the cartilage secretome. MMP-3 is a proteolytic enzyme that degrades ECM components such as fibronectin, collagens and cartilage proteoglycans. Its levels are increased in clinical OA cases and the gene encoding this protein has been shown to be up-regulated in IL-1β-stimulated chondrocytes *in vitro* [45,46]*.* Immunohistochemical studies have reported MMP-3 in the synovium, cartilage and medium of cultured cartilage explants from OA patients [13]. Our qualitative LC–MS/MS data suggested that MMP-3 was higher in IL-1β-treated samples compared to controls and this observation was confirmed by western blotting. NSAID-treatment actually reduced MMP-3 levels, an observation that has been found previously for bovine MMP-3 mRNA and protein [47]. The weak band(s) in the tissue homogenate controls showed that MMP-3 was present in cartilage, but in lower concentrations than in the IL-1β-treated samples and thus may have been below the detection threshold used to analyse the samples.

Western blotting for MMP-1 detected a band of roughly 102 kDa in both the cartilage control and all the secretome samples (present as a double band in IL-1β-treated samples corresponding to active and inactive forms), which was nearly double the predicted molecular weight (54 kDa). Although the samples were run under reducing conditions, it may be that the observed band corresponds to a dimer form of MMP-1 that was resistant to the reducing agent.

The equine sequence for TSP-1 was not present in the Swiss-Prot database at the time of this study but cross species matching with bovine, human and mouse confirmed the presence of TSP-1. The fragments matched the protein motifs of TSP-1 from multiple other species, thus increasing our confidence in the detection of this protein in the horse cartilage secretome. The presence of TSP-1 was also confirmed by western blotting using two different commercial antibodies. TSP-1 is an adhesive glycoprotein that mediates cell-to-cell and cell-to-matrix interactions [48,49]. Human cartilage studies have shown that TSP-1 is increased in early OA cartilage lesions [50] and both the proteomic and western blot results from our study, suggest that this is reflected in our cartilage explant model of early OA.

The abundance of cartilage ECM-associated proteins in the top scoring entries highlights the specificity of this *in vitro* model and the suitability of high throughput protein discovery techniques for interrogating it. However, despite using a targeted approach and an explant culture system, a small number of non-matrix associated proteins were also identified in the media of several samples, for example, vimentin, dynein heavy chain, alpha enolase and the membrane bound sodium hydrogen exchanger 2 (NHE2) (see Tables 2 and 3). There are a number of possible explanations and interpretations for the presence of these proteins. The most obvious explanation for the presence of these non-ECM proteins is the high sensitivity of the high-throughput approach for detecting proteins released from the cytoplasm following cell death; the detection of these proteins may be attributable to a small number of dead or dying chondrocytes. However, it is known that there are frequently observed proteins in many proteomic experiments regardless of species or tissue being studied, such as alpha enolase and vimentin [51,52].

In addition to the cartilage ECM and chondrocyte-associated proteins, the database search also detected some apparently unlikely candidates for biomarkers of arthritis, for example Bullous pemphigoid antigen or breast cancer type 2 protein susceptibility homologue. However, it is highly likely that some of these less abundant proteins with lower Mowse scores could be proteins of interest in their own right or modifications of the abundant proteins, which are relevant to OA. Ideal biomarkers for OA are those that can indicate the very early stages of disease before too much destruction has occurred [53]. Therefore the best markers of early OA are unlikely to be fragments or degraded forms of major ECM proteins such as fibrillar collagens [54]. Notably, we did not identify any fibrillar collagens in this study. This adds weight to the argument that incubating cartilage explants with IL-1β creates an environment that could mimic the early stages of OA. Although we used a Mowse score of 40 as the cut-off point, it is possible that proteins under the detection threshold could be potentially useful biomarkers. For example, the protein arginine deiminase type-4 (PADI4), which had a Mowse score of 39 (see Table 4), is known to catalyse citrullination of arginine residues of proteins. Citrullinated peptides can act as autoantigens thereby increasing the risk of rheumatoid arthritis (RA) [55], as such, PADI4 gene expression has been detected in the synovial tissue from RA patients [56]. In addition, autoantibodies against PADI4 are associated with increased RA severity [57]. Although RA is an auto-immune disease, unlike OA, the protein may have other functions and potential involvement in OA. The presence of PADI4 in the supernatant of the cartilage explants in this study may partly explain why this enzyme could be a target of autoantibodies to citrullinated proteins, in fact this antigen may not be as specific for rheumatoid arthritis (RA) as previously suggested [58,59], the enzyme may also be released in OA. PADI4 is one of the many less abundant proteins identified in the model that may be potential biomarkers. More studies with larger sample numbers may provide more information in this area. However, greater potential lies in using methods that facilitate mining below the abundant proteins in the secretome to allow robust and clear detection of the lower abundance components. The most obvious of these methods would be the depletion of the major/high abundance proteins, which is commonly used when studying biological fluids such as plasma and cerebrospinal fluid [60,61]. Another approach would be the use of metabolic labelling during culture to allow the newly synthesised proteins to be studied [62]. Adopting these approaches will aid identification of these less abundant but potentially more interesting proteins in terms of the molecular changes accompanying early OA.

Proteomics has been used to study soluble extracts of cartilage [24,63], chondrocyte whole cell lysates [64], synovial fluid [65] and synovial fibroblasts [66], from both normal and OA patients. More recently proteomics has been used to characterise the extracellular space in tracheal in the human aorta [67]. The cartilage explant model employed in our study can reduce some of the confounding variables associated with clinical samples such as individual variation, differing disease aetiology and the stage of disease progression. In effect, using the cartilage explant system provides a more homogenous model with appropriate controls. In addition, explants can be cultured in serum-free media, which address the technical issue of contaminating serum albumins and other high abundance proteins, which can prevent the identification of the lower abundance proteins.

The advantage of a proteomic approach over a genomic approach to studying potential disease indicators are that gene expression levels do not necessarily correlate with protein levels [68]. For example, CLU expression is increased at the mRNA level in early OA cartilage and decreased in advanced OA [69]. IL-1α-stimulated cartilage explants have been shown to produce decreased levels of CLU compared to untreated cartilage [25]. This could reflect the differences between clinical cases and *in vitro* models of OA and the various stimuli used to initiate cartilage degradation. Clearly, further work is needed to look at levels of these proteins in the synovial fluid and serum of clinical cases.

## 5 Conclusions

This study refined an existing explant model of equine articular cartilage and adapted it for subsequent high throughput proteomic work. One of the key advantages of this cartilage explant model is that it is serum-free, so the problem of contaminating serum proteins such as albumins and immunoglobulins, which make up a large percentage of serum, is eliminated using this approach. Another advantage of this culture system is that the resident cells are maintained in their native microenvironment overcoming the difficulties and challenges associated with chondrocyte dedifferentiation in proteomic studies that use primary cultured cells *in vitro* [70]*.* Cross species peptide matching identified a number of relevant proteins with well-established ECM functions in cartilage including: cell–matrix and matrix–matrix interactions (fibronectin, TSP-1, COMP, CHAD); matrix turnover (MMP-1, MMP-3) and extracellular molecular chaperone activity (CLU). Other proteomic studies using cartilage from other species have identified similar panels of ECM proteins [24,25,28]. Equine cartilage has not been examined previously in this way and comparisons with the mouse, human and bovine cartilage proteomes also substantiate data obtained in those previous reports. These observations confirm and complement previous studies in other species and are good indicators for the usefulness of both the explant model of cartilage inflammation and the high throughput methodology used. It is important to point out that the basic biology of articular cartilage and the molecular mechanisms involved in its degradation in arthritis are essentially the same in all mammalian species. Any reported differences observed between different species could be due to different pathogenic stimuli, the root aetiologies of the disease and the biological reagents used in the experiments. Therefore, given the many similarities that exist between equine and human cartilage in the biological context, the results of this study could be used for understanding disease processes in both species.

Our novel approach justifies the application of high throughput techniques in basic cartilage biology and OA research. Furthermore, this strategy may identify clinically useful secreted biomarkers for identifying structural and biochemical changes in early stages of OA. The explant culture system employed also facilitated a surprisingly rapid and ‘clean’ analysis of secreted proteins in our *in vitro* model of cartilage inflammation. This was an important and novel element of this study. Furthermore, the horse is a large animal model that may serve as a surrogate for human OA and may eventually lead to the discovery of candidate biomarkers. Future studies will exploit this serum-free model to investigate the effects of other pro-inflammatory cytokines and pathophysiologically relevant catabolic stimuli such as mechanical injury [71].

## Competing interests

This paper was written by the authors within the scope of their academic and research positions. The authors declare that they have no competing interests.

## Authors' contributions

All authors have made substantial intellectual contributions to the conception and design of the study, data acquisition, analysis and interpretation. ALC carried out the *in vitro* experimental work and contributed to data collection and interpretation and analysis. JRS carried out the high-throughput proteomic analysis at Bruker UK. AM and JRS conceived the study design, coordinated the experiments and contributed to data interpretation and manuscript preparation with the collaboration of SL, DA and PH. SL made a significant contribution to the strategic development of the study, MS data analysis and interpretation as well as submission of the MS data to the PRIDE database. All authors have approved the final version submitted.

## Figures and Tables

**Table 1:** Secreted extracellular matrix proteins and proteolytic enzymes present in multiple samples as identified using the SwissProt 51.6 database.

Protein name	Species matched	Accession number	SwissProt entry name	Mascot Mowse score	Sequence coverage (%)	No. of matched peptides
Fibronectin	Mouse, cow, rat, horse	P07589	FINC_BOVIN	642	9	16
Cartilage oligomeric matrix protein	Human, mouse, cow, rat	Q9R0G6	COMP_MOUSE	554	16	11
Matrix-metalloproteinase-3	Horse	Q28397	MMP3_HORSE	366	19	11
Aggrecan core protein	Human, mouse, rat, pig, dog	P16112	PGCA_HUMAN	353	3	8
Clusterin	Horse, dog	Q29482	CLUS_HORSE	318	14	5
Thrombospondin-1	Human, mouse, cow	P07996	TSP1_HUMAN	227	5	6
Matrix-metalloproteinase-1	Horse, cow	Q9XSZ5	MMP1_HORSE	227	10	5
Fibronectin	Mouse, cow, rat, horse	Q28377	FINC_HORSE	221	11	3
Chondroadherin	Human, mouse, cow, rat	O15335	CHAD_HUMAN	220	13	4
Biglycan	Horse, cow	O46403	PGS1_HORSE	183	13	4
Decorin	Human, horse, cow, rat, dog, rabbit	O46542	PGS2_HORSE	156	10	3
Cartilage intermediate layer protein−1	Human, mouse, pig	O75339	CILP1_HUMAN	99	2	3
Prolargin precursor	Human, mouse	P51888	PRELP_HUMAN	96	3	1
TNF receptor 11B precursor	Mouse	O08712	TR11B_MOUSE	87	3	1
Fibromodulin	Human, mouse, cow, rat, horse	P50609	FMOD_RAT	82	8	3

**Table 2:** Non-extracellular matrix proteins identified in six or more samples as identified using the SwissProt 51.6 database.

Protein name	Species matched	Accession number	SwissProt entry name	Mascot Mowse score	Sequence coverage (%)	No. of matched peptides
Vimentin	Cow, green monkey	P48616	VIME_BOVIN	301	12	6
Serum amyloid A protein	Cow, horse	P19857	SAA_HORSE	123	25	3
Alpha Enolase	Human, cow	Q9XSJ4	ENOA_BOVIN	94	7	4
Serum amyloid A2 protein	American mink	P02739	SAA2_MUSVI	74	12	2
Transcription termination factor 1	Human	Q15361	TTF1_HUMAN	68	2	3
Serum albumin	Cow	P02769	ALBU_BOVIN	71	1	1
Breast cancer type 2 protein susceptibility homolog	Cat	Q864S8	BRCA2_FELCA	51	0	3
Lysozyme C, kidney isozyme	Sheep	P80190	LYSCK_SHEEP	47	6	1
Sodium/hydrogen exchanger 2	Rabbit	P50482	SL9A2_RABBIT	45	1	1
Cytoplasmic dynein 1 heavy chain 1	Human	Q14204	DYHC1_HUMAN	44	0	1

**Table 3:** Non-extracellular matrix proteins identified in only one sample. The identifications of these proteins relied on peptides with four or more continuous fragment ion matches in a row using the SwissProt 51.6 database.

Protein name	Species matched	Accession number	SwissProt entry name	Mascot Mowse score	Sequence coverage (%)	No. of matched peptides	No. of continuous fragment ions in a series
Keratin, type II cytoskeletal 1	Human	P04264	K2C1_HUMAN	87	3	2	9
Triosephosphate isomerase	Dog	P54714	TPIS_CANFA	52	4	1	9
Fc receptor-like A	Mouse	Q920A9	FCRLA_MOUSE	49	1	1	5
Glucose-6-phosphate 1-dehydrogenase 2	Mouse	P97324	G6PD2_MOUSE	45	3	2	4
Centromere protein C1	Mouse	P49452	CENPC_MOUSE	42	0	1	4
Lactadherin	Pig	P79385	MFGM_PIG	40	2	1	4
Max-like protein X	Human	Q9UH92	MLX_HUMAN	38	2	1	4

**Table 4:** Non-extracellular matrix proteins identified in only one sample. The identifications of these proteins relied on peptides with fewer than 4 continuous fragment ion matches in a row using the SwissProt 51.6 database.

Protein name	Species matched	Accession number	SwissProt entry name	Mascot Mowse score	Sequence coverage (%)	No. of matched peptides
DNA-dependent protein kinase catalytic subunit	Dog	Q8WN22	PRKDC_CANFA	55	0	3
Serine-protein kinase ATM	Mouse	Q62388	ATM_MOUSE	55	0	3
Histone acetyltransferase	Human	Q8WYB5	MYST4_HUMAN	53	1	2
Bromodomain and PHD finger-containing protein 3	Human	Q9ULD4	BRPF3_HUMAN	51	2	3
Ubiquitin carboxyl-terminal hydrolase 24	Human	Q9UPU5	UBP24_HUMAN	50	0	2
Putative pre-mRNA-splicing factor ATP dependent RNA helicase DXH15	Human	O43143	DHX15_HUMAN	48	2	2
Nuclear mitotic apparatus protein 1	Human	Q14980	NUMA1_HUMAN	48	0	2
Bullous pemphigoid antigen 1	Human	Q03001	BPA1_HUMAN	47	0	2
Sodium/calcium exchanger 1	Cow	P48765	NAC1_BOVIN	47	1	2
Thyroid receptor-interacting protein-11	Human	Q15643	TRIPB_HUMAN	47	0	1
Plectin-1	Rat	P30427	PLEC1_RAT	46	0	3
Bcl-2/adenovirus E1B 19 kDa-interacting protein 2-like protein	Mouse	Q99JU7	BNIPL_MOUSE	45	6	1
Epidermal growth factor receptor kinase substrate 8-like protein 3	Human	Q8TE67	ES8L3_HUMAN	44	2	2
Glutaminyl-tRNA synthetase	Human	P47897	SYQ_HUMAN	44	2	2
Integrin beta-5	Human	P18084	ITB5_HUMAN	43	1	2
Glial fibrillary acidic protein	Cow	Q28115	GFAP_BOVIN	43	5	2
Phosphoglycerate kinase 1	Chinese Hamster	P50310	PGK1_CRIGR	43	2	1
Synergin gamma (AP1 subunit gamma-binding protein 1)	Mouse	Q5SV85	SYNRG_MOUSE	42	1	2
Beta, beta carotene 9′,10′-oxygenase	Crab eating macaque	Q8HXG8	BCDO2_MACFA	42	3	2
Death-associated protein kinase 3	Rat	O88764	DAPK3_RAT	41	2	1
Isocitrate dehydrogenase [NAD] subunit beta, mitochondrial	Cow	O77784	IDH3B_BOVIN	41	1	1
Cytosolic phospholipase A2 delta	Mouse	Q50L43	PA24D_MOUSE	40	2	2
Protein arginine deiminase type-4	Human	Q9UM07	PADI4_HUMAN	39	1	2

## Appendix A Supplementary data

Supplementary material 2.

How to reference PRIDE submissions.

## Footnotes

1 Mowse scoring algorithm http://www.matrixscience.com/help/scoring_help.html#MOW.

## References

[1] Buckwalter J.A., Mankin H.J., Grodzinsky A.J. (2005). Articular cartilage and osteoarthritis. Instr Course Lect.

[2] Buckwalter J.A., Mankin H.J. (1998). Articular cartilage: tissue design and chondrocyte–matrix interactions. Instr Course Lect.

[3] Eyre D.R. (2004). Collagens and cartilage matrix homeostasis. Clin Orthop Relat Res.

[4] Kuettner K.E. (1992). Biochemistry of articular cartilage in health and disease. Clin Biochem.

[5] Hardingham T.E., Fosang A.J. (1992). Proteoglycans: many forms and many functions. FASEB J.

[6] Feng H., Danfelter M., Stromqvist B., Heinegard D. (2006). Extracellular matrix in disc degeneration. J Bone Joint Surg Am.

[7] Archer C.W., Francis-West P. (2003). The chondrocyte. Int J Biochem Cell Biol.

[8] Goldring M.B., Marcu K.B. (2009). Cartilage homeostasis in health and rheumatic diseases. Arthritis Res Ther.

[9] Aigner T., Soeder S., Haag J. (2006). IL-1beta and BMPs—interactive players of cartilage matrix degradation and regeneration. Eur Cell Mater.

[10] Goldring M.B., Goldring S.R. (2007). Osteoarthritis. J Cell Physiol.

[11] Samuels J., Krasnokutsky S., Abramson S.B. (2008). Osteoarthritis: a tale of three tissues. Bull NYU Hosp Jt Dis.

[12] Felson D.T. (2004). An update on the pathogenesis and epidemiology of osteoarthritis. Radiol Clin North Am.

[13] Okada Y., Shinmei M., Tanaka O., Naka K., Kimura A., Nakanishi I. (1992). Localization of matrix metalloproteinase 3 (stromelysin) in osteoarthritic cartilage and synovium. Lab Invest.

[14] Goldring S.R., Goldring M.B. (2004). The role of cytokines in cartilage matrix degeneration in osteoarthritis. Clin Orthop Relat Res.

[15] Goldring M.B., Berenbaum F. (2004). The regulation of chondrocyte function by proinflammatory mediators: prostaglandins and nitric oxide. Clin Orthop Relat Res.

[16] Aigner T., Vornehm S.I., Zeiler G., Dudhia J., von der Mark K., Bayliss M.T. (1997). Suppression of cartilage matrix gene expression in upper zone chondrocytes of osteoarthritic cartilage. Arthritis Rheum.

[17] Clark A.G., Jordan J.M., Vilim V., Renner J.B., Dragomir A.D., Luta G. (1999). Serum cartilage oligomeric matrix protein reflects osteoarthritis presence and severity: the Johnston County Osteoarthritis Project. Arthritis Rheum.

[18] Rousseau J.C., Delmas P.D. (2007). Biological markers in osteoarthritis. Nat Clin Pract Rheumatol.

[19] Williams F.M. (2009). Biomarkers: in combination they may do better. Arthritis Res Ther.

[20] Polacek M., Bruun J.A., Johansen O., Martinez I. (2010). Differences in the secretome of cartilage explants and cultured chondrocytes unveiled by SILAC technology. J Orthop Res.

[21] Ruiz-Romero C., Calamia V., Carreira V., Mateos J., Fernandez P., Blanco F.J. (2010). Strategies to optimize two-dimensional gel electrophoresis analysis of the human joint proteome. Talanta.

[22] Ruiz-Romero C., Lopez-Armada M.J., Blanco F.J. (2005). Proteomic characterization of human normal articular chondrocytes: a novel tool for the study of osteoarthritis and other rheumatic diseases. Proteomics.

[23] Ruiz-Romero C., Blanco F.J. (2010). Proteomics role in the search for improved diagnosis, prognosis and treatment of osteoarthritis. Osteoarthritis Cartilage.

[24] Wu J., Liu W., Bemis A., Wang E., Qiu Y., Morris E.A. (2007). Comparative proteomic characterization of articular cartilage tissue from normal donors and patients with osteoarthritis. Arthritis Rheum.

[25] Wilson R., Belluoccio D., Little C.B., Fosang A.J., Bateman J.F. (2008). Proteomic characterization of mouse cartilage degradation in vitro. Arthritis Rheum.

[26] Wilson R., Belluoccio D., Little C.B., Fosang A.J., Bateman J.F. (2008). Proteomic characterization of mouse cartilage degradation in vitro. Arthritis Rheum.

[27] Cillero-Pastor B., Ruiz-Romero C., Carames B., Lopez-Armada M.J., Blanco F.J. (2010). Proteomic analysis by two-dimensional electrophoresis to identify the normal human chondrocyte proteome stimulated by tumor necrosis factor alpha and interleukin-1beta. Arthritis Rheum.

[28] Stevens A.L., Wishnok J.S., Chai D.H., Grodzinsky A.J., Tannenbaum S.R. (2008). A sodium dodecyl sulfate-polyacrylamide gel electrophoresis-liquid chromatography tandem mass spectrometry analysis of bovine cartilage tissue response to mechanical compression injury and the inflammatory cytokines tumor necrosis factor alpha and interleukin-1beta. Arthritis Rheum.

[29] Li H., Yang H.S., Wu T.J., Zhang X.Y., Jiang W.H., Ma Q.L. (2010). Proteomic analysis of early-response to mechanical stress in neonatal rat mandibular condylar chondrocytes. J Cell Physiol.

[30] Zhang W.B., Wang L. (2009). Label-free quantitative proteome analysis of skeletal tissues under mechanical load. J Cell Biochem.

[31] Perkins D.N., Pappin D.J., Creasy D.M., Cottrell J.S. (1999). Probability-based protein identification by searching sequence databases using mass spectrometry data. Electrophoresis.

[32] Vizcaíno J.A., Côté R., Reisinger F., Foster J.M., Mueller M., Rameseder J. (2009). A guide to the Proteomics Identifications Database proteomics data repository. Proteomics.

[33] Barsnes H., Vizcaino J.A., Eidhammer I., Martens L. (2009). PRIDE Converter: making proteomics data-sharing easy. Nat Biotechnol.

[34] Lester P.J., Hubbard S.J. (2002). Comparative bioinformatic analysis of complete proteomes and protein parameters for cross-species identification in proteomics. Proteomics.

[35] Liska A.J., Shevchenko A. (2003). Expanding the organismal scope of proteomics: cross-species protein identification by mass spectrometry and its implications. Proteomics.

[36] Chamrad D.C., Koerting G., Gobom J., Thiele H., Klose J., Meyer H.E. (2003). Interpretation of mass spectrometry data for high-throughput proteomics. Anal Bioanal Chem.

[37] Clutterbuck A.L., Mobasheri A., Shakibaei M., Allaway D., Harris P. (2009). Interleukin-1beta-induced extracellular matrix degradation and glycosaminoglycan release is inhibited by curcumin in an explant model of cartilage inflammation. Ann NY Acad Sci.

[38] DiCesare P.E., Morgelin M., Mann K., Paulsson M. (1994). Cartilage oligomeric matrix protein and thrombospondin 1. Purification from articular cartilage, electron microscopic structure, and chondrocyte binding. Eur J Biochem.

[39] Rosenberg K., Olsson H., Morgelin M., Heinegard D. (1998). Cartilage oligomeric matrix protein shows high affinity zinc-dependent interaction with triple helical collagen. J Biol Chem.

[40] Lohmander L.S., Saxne T., Heinegard D.K. (1994). Release of cartilage oligomeric matrix protein (COMP) into joint fluid after knee injury and in osteoarthritis. Ann Rheum Dis.

[41] Misumi K., Tagami M., Kamimura T., Miyakoshi D., Helal I.E., Arai K. (2006). Urine cartilage oligomeric matrix protein (COMP) measurement is useful in discriminating the osteoarthritic Thoroughbreds. Osteoarthritis Cartilage.

[42] Arai K., Misumi K., Carter S.D., Shinbara S., Fujiki M., Sakamoto H. (2005). Analysis of cartilage oligomeric matrix protein (COMP) degradation and synthesis in equine joint disease. Equine Vet J.

[43] Petersson I.F., Boegard T., Dahlstrom J., Svensson B., Heinegard D., Saxne T. (1998). Bone scan and serum markers of bone and cartilage in patients with knee pain and osteoarthritis. Osteoarthritis Cartilage.

[44] Tseng S., Reddi A.H., Di Cesare P.E. (2009). Cartilage oligomeric matrix protein (COMP): a biomarker of arthritis. Biomark Insights.

[45] Brama P.A., TeKoppele J.M., Beekman B., van El B., Barneveld A., van Weeren P.R. (2000). Influence of development and joint pathology on stromelysin enzyme activity in equine synovial fluid. Ann Rheum Dis.

[46] Tung J.T., Fenton J.I., Arnold C., Alexander L., Yuzbasiyan-Gurkan V., Venta P.J. (2002). Recombinant equine interleukin-1beta induces putative mediators of articular cartilage degradation in equine chondrocytes. Can J Vet Res.

[47] Sadowski T., Steinmeyer J. (2001). Effects of non-steroidal antiinflammatory drugs and dexamethasone on the activity and expression of matrix metalloproteinase-1, matrix metalloproteinase-3 and tissue inhibitor of metalloproteinases-1 by bovine articular chondrocytes. Osteoarthritis Cartilage.

[48] Lawler J., Hynes R.O. (1986). The structure of human thrombospondin, an adhesive glycoprotein with multiple calcium-binding sites and homologies with several different proteins. J Cell Biol.

[49] Chen H., Herndon M.E., Lawler J. (2000). The cell biology of thrombospondin-1. Matrix Biol.

[50] Pfander D., Cramer T., Deuerling D., Weseloh G., Swoboda B. (2000). Expression of thrombospondin-1 and its receptor CD36 in human osteoarthritic cartilage. Ann Rheum Dis.

[51] Petrak J., Ivanek R., Toman O., Cmejla R., Cmejlova J., Vyoral D. (2008). Deja vu in proteomics. A hit parade of repeatedly identified differentially expressed proteins. Proteomics.

[52] Wang P., Bouwman F.G., Mariman E.C. (2009). Generally detected proteins in comparative proteomics—a matter of cellular stress response?. Proteomics.

[53] Kraus V.B. (2005). Biomarkers in osteoarthritis. Curr Opin Rheumatol.

[54] Mobasheri A., Henrotin Y., Lawler J. (2010). Identification, validation and qualification of biomarkers for osteoarthritis in humans and companion animals: mission for the next decade. Vet J.

[55] Vossenaar E.R., Despres N., Lapointe E., van der Heijden A., Lora M., Senshu T. (2004). Rheumatoid arthritis specific anti-Sa antibodies target citrullinated vimentin. Arthritis Res Ther.

[56] Suzuki A., Yamada R., Chang X., Tokuhiro S., Sawada T., Suzuki M. (2003). Functional haplotypes of PADI4, encoding citrullinating enzyme peptidylarginine deiminase 4, are associated with rheumatoid arthritis. Nat Genet.

[57] Harris M.L., Darrah E., Lam G.K., Bartlett S.J., Giles J.T., Grant A.V. (2008). Association of autoimmunity to peptidyl arginine deiminase type 4 with genotype and disease severity in rheumatoid arthritis. Arthritis Rheum.

[58] Kolfenbach J.R., Deane K.D., Derber L.A., O'Donnell C.I., Gilliland W.R., Edison J.D. (2010). Autoimmunity to peptidyl arginine deiminase type 4 precedes clinical onset of rheumatoid arthritis. Arthritis Rheum.

[59] Auger I., Martin M., Balandraud N., Roudier J. (2010). Rheumatoid arthritis-specific autoantibodies to peptidyl arginine deiminase type 4 inhibit citrullination of fibrinogen. Arthritis Rheum.

[60] Tirumalai R.S., Chan K.C., Prieto D.A., Issaq H.J., Conrads T.P., Veenstra T.D. (2003). Characterization of the low molecular weight human serum proteome. Mol Cell Proteomics.

[61] Shores K.S., Knapp D.R. (2007). Assessment approach for evaluating high abundance protein depletion methods for cerebrospinal fluid (CSF) proteomic analysis. J Proteome Res.

[62] Zwickl H., Traxler E., Staettner S., Parzefall W., Grasl-Kraupp B., Karner J. (2005). A novel technique to specifically analyze the secretome of cells and tissues. Electrophoresis.

[63] Guo D., Tan W., Wang F., Lv Z., Hu J., Lv T. (2008). Proteomic analysis of human articular cartilage: identification of differentially expressed proteins in knee osteoarthritis. Joint Bone Spine.

[64] Ruiz-Romero C., Carreira V., Rego I., Remeseiro S., Lopez-Armada M.J., Blanco F.J. (2008). Proteomic analysis of human osteoarthritic chondrocytes reveals protein changes in stress and glycolysis. Proteomics.

[65] Gobezie R., Kho A., Krastins B., Sarracino D.A., Thornhill T.S., Chase M. (2007). High abundance synovial fluid proteome: distinct profiles in health and osteoarthritis. Arthritis Res Ther.

[66] Bo G.P., Zhou L.N., He W.F., Luo G.X., Jia X.F., Gan C.J. (2009). Analyses of differential proteome of human synovial fibroblasts obtained from arthritis. Clin Rheumatol.

[67] Didangelos A., Yin X., Mandal K., Baumert M., Jahangiri M., Mayr M. (2010). Proteomics characterization of extracellular space components in the human aorta. Mol Cell Proteomics.

[68] Nie L., Wu G., Culley D.E., Scholten J.C., Zhang W. (2007). Integrative analysis of transcriptomic and proteomic data: challenges, solutions and applications. Crit Rev Biotechnol.

[69] Connor J.R., Kumar S., Sathe G., Mooney J., O'Brien S.P., Mui P. (2001). Clusterin expression in adult human normal and osteoarthritic articular cartilage. Osteoarthritis Cartilage.

[70] von der Mark K., Gauss V., von der Mark H., Muller P. (1977). Relationship between cell shape and type of collagen synthesised as chondrocytes lose their cartilage phenotype in culture. Nature.

[71] Quinn T.M., Grodzinsky A.J., Hunziker E.B., Sandy J.D. (1998). Effects of injurious compression on matrix turnover around individual cells in calf articular cartilage explants. J Orthop Res.

