# Supplementary figures and images for: High throughput proteomic analysis of the secretome in an explant model of articular cartilage inflammation
Source: J Proteomics. 2011 May 1;74(5-2):704–15. doi: 10.1016/j.jprot.2011.02.017 (PMC3078332; doi:10.1016/j.jprot.2011.02.017)

## Slide 1
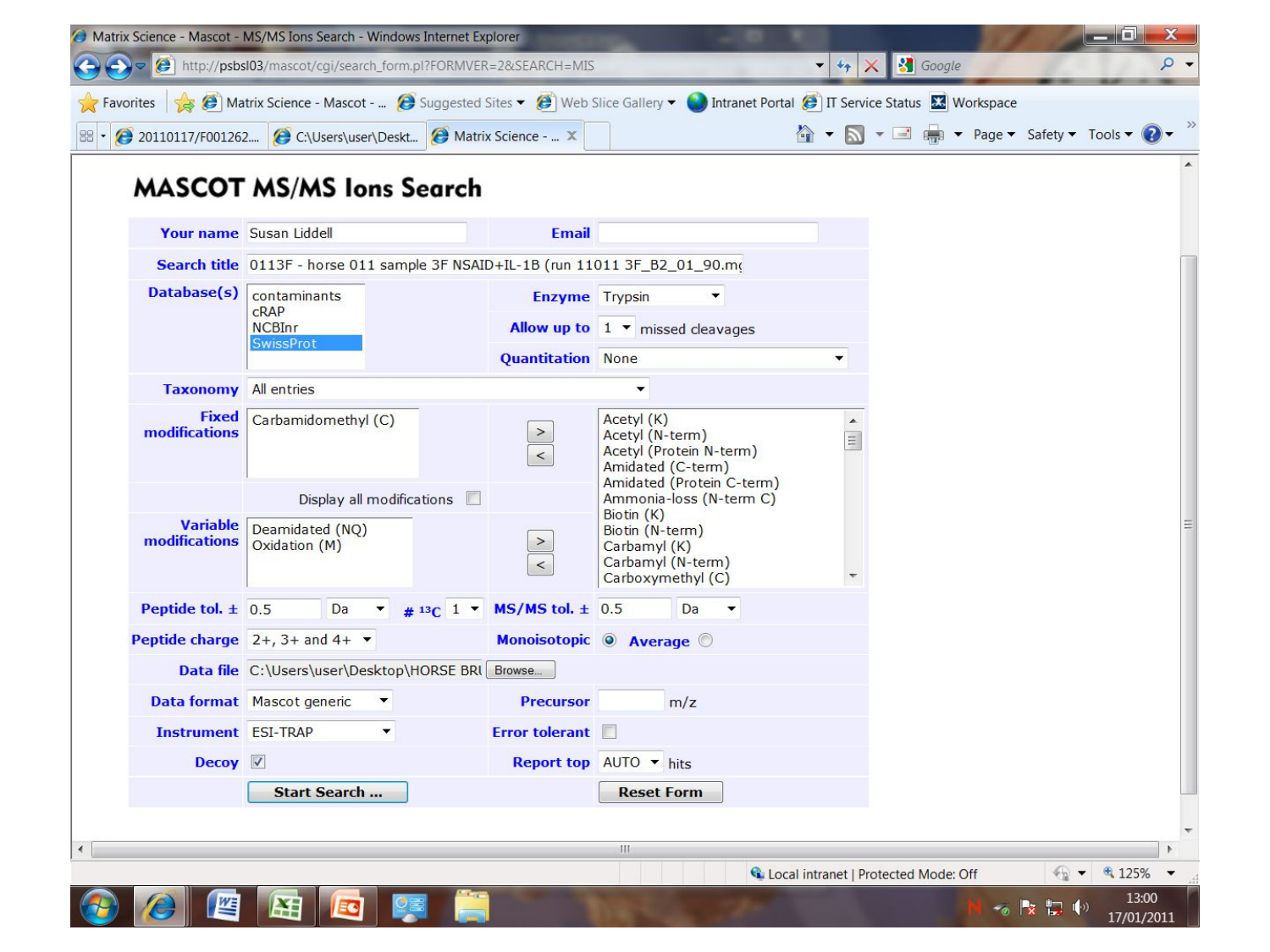

#

Supplement: Supplementary file 1 — Supplementary material 1. [file mmc1.ppt]
