# Supplementary material for: High throughput proteomic analysis of the secretome in an explant model of articular cartilage inflammation
Source: J Proteomics. 2011 May 1;74(5-2):704–15. doi: 10.1016/j.jprot.2011.02.017 (PMC3078332; doi:10.1016/j.jprot.2011.02.017)

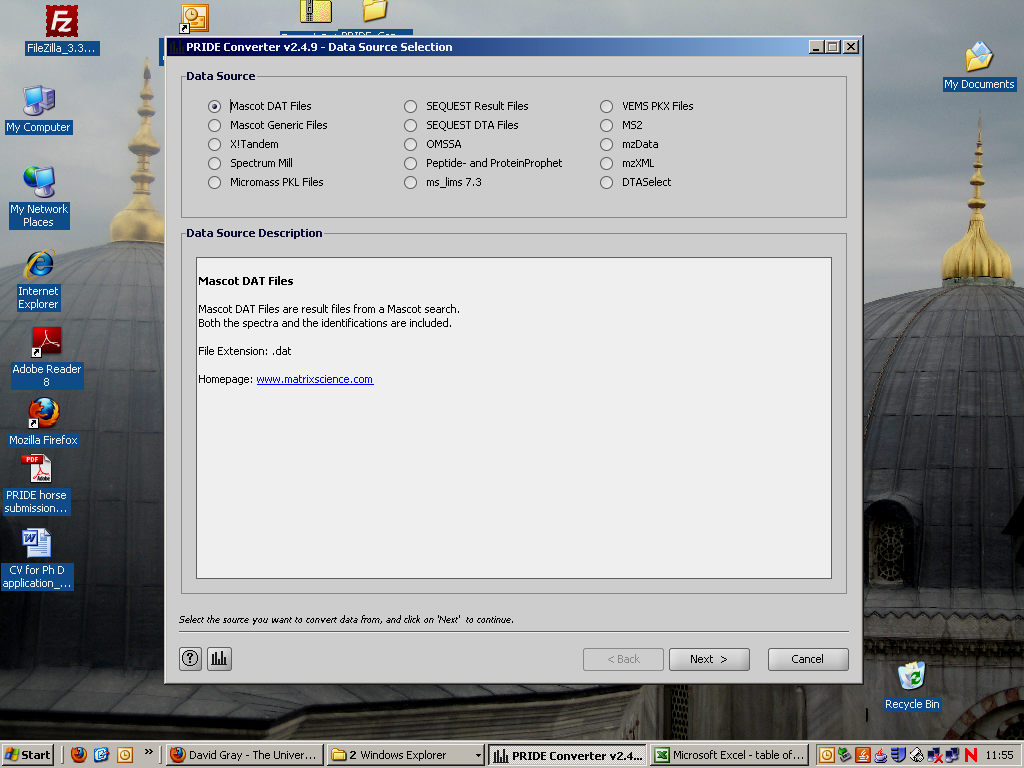


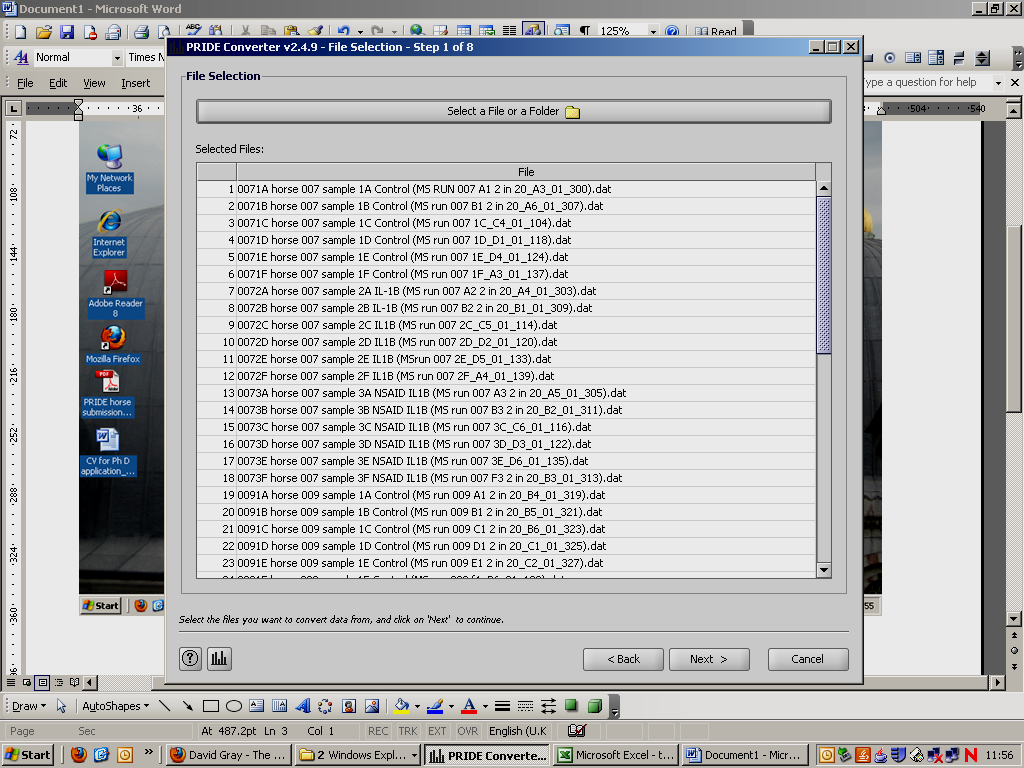


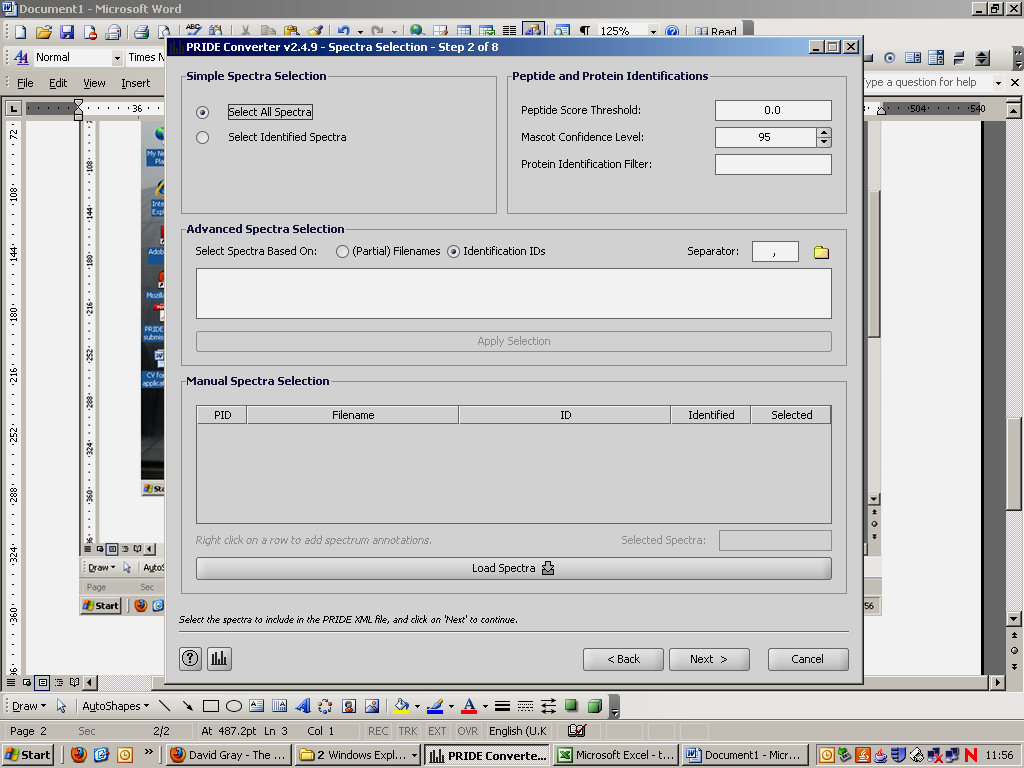


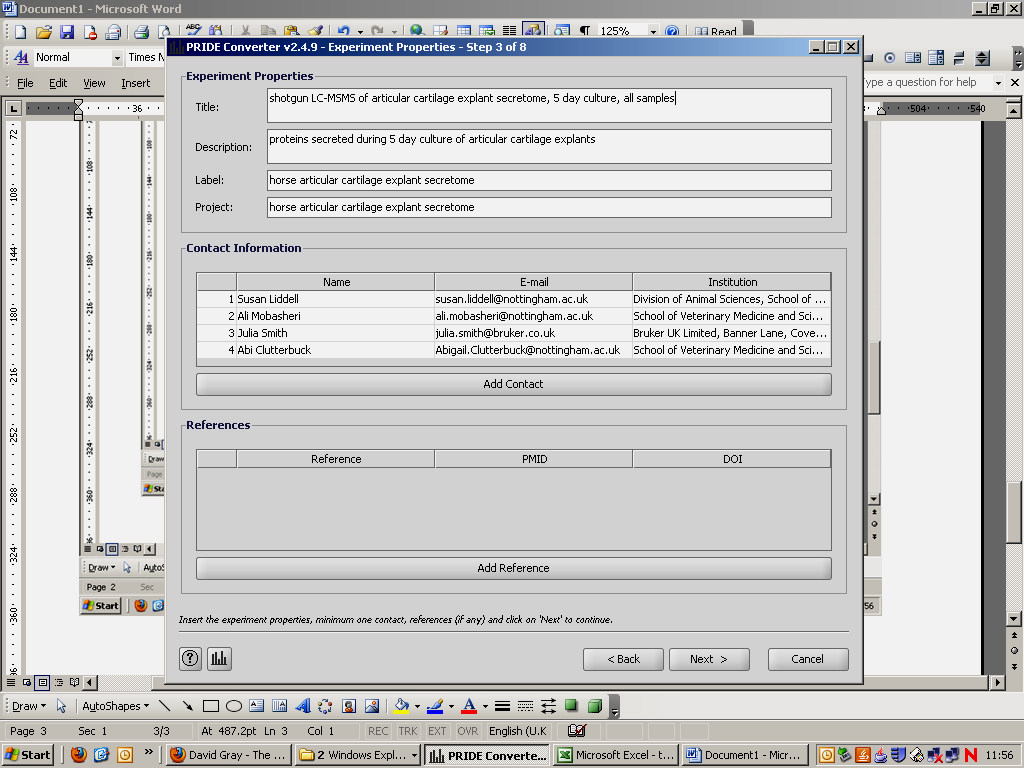


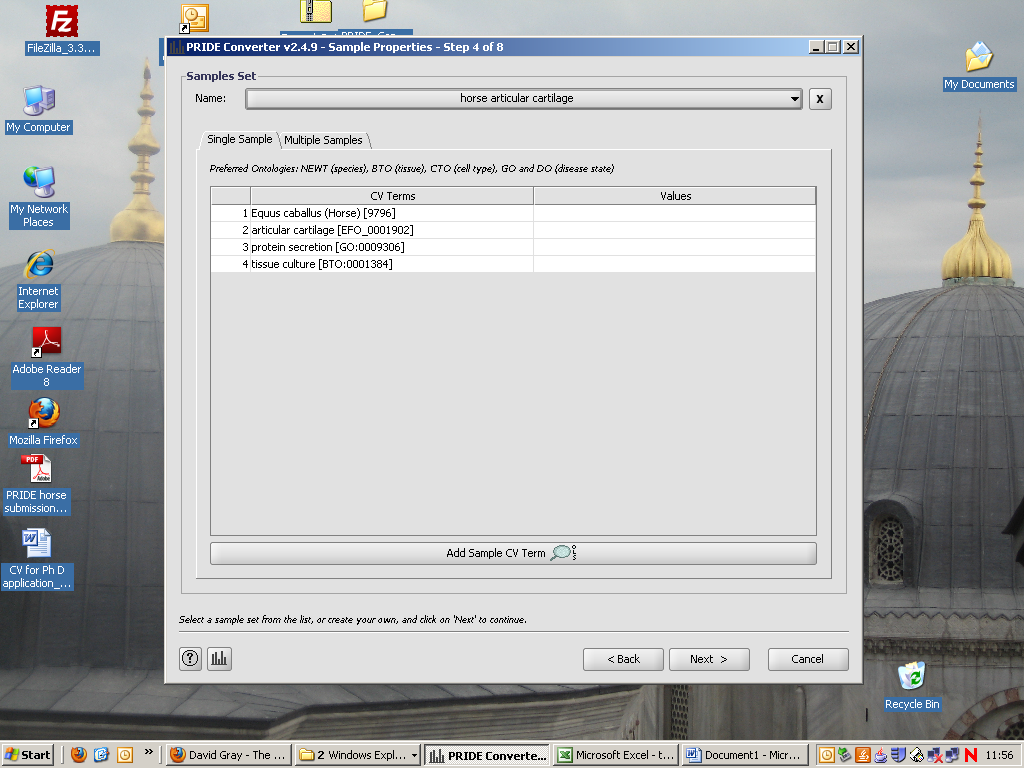


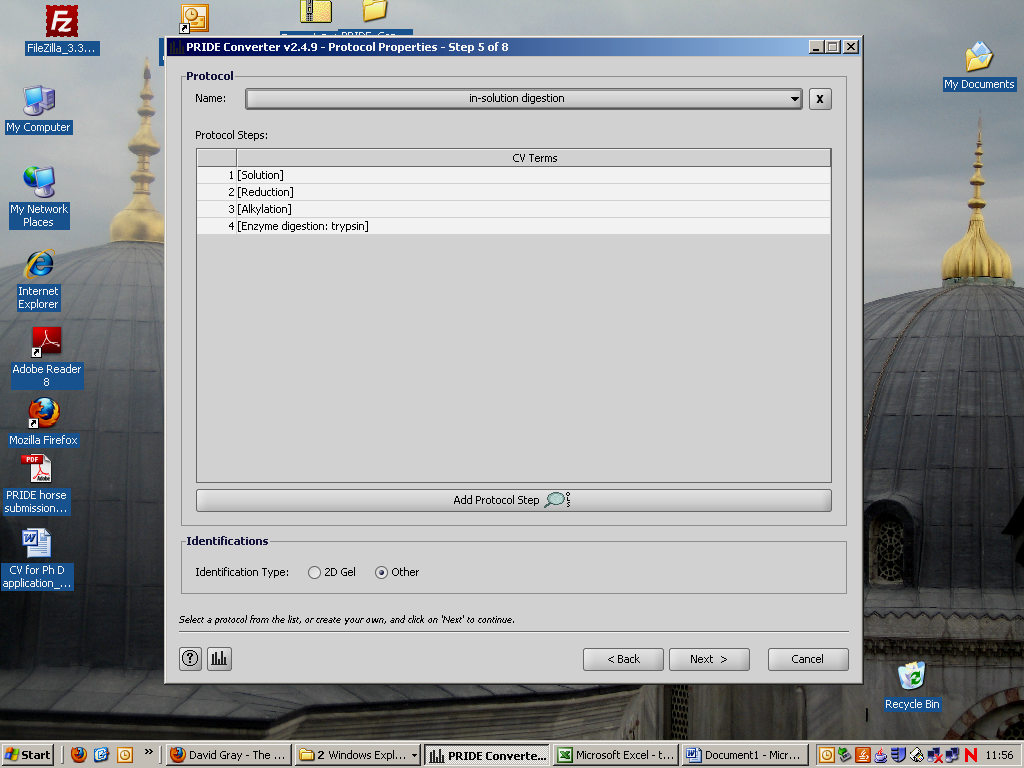


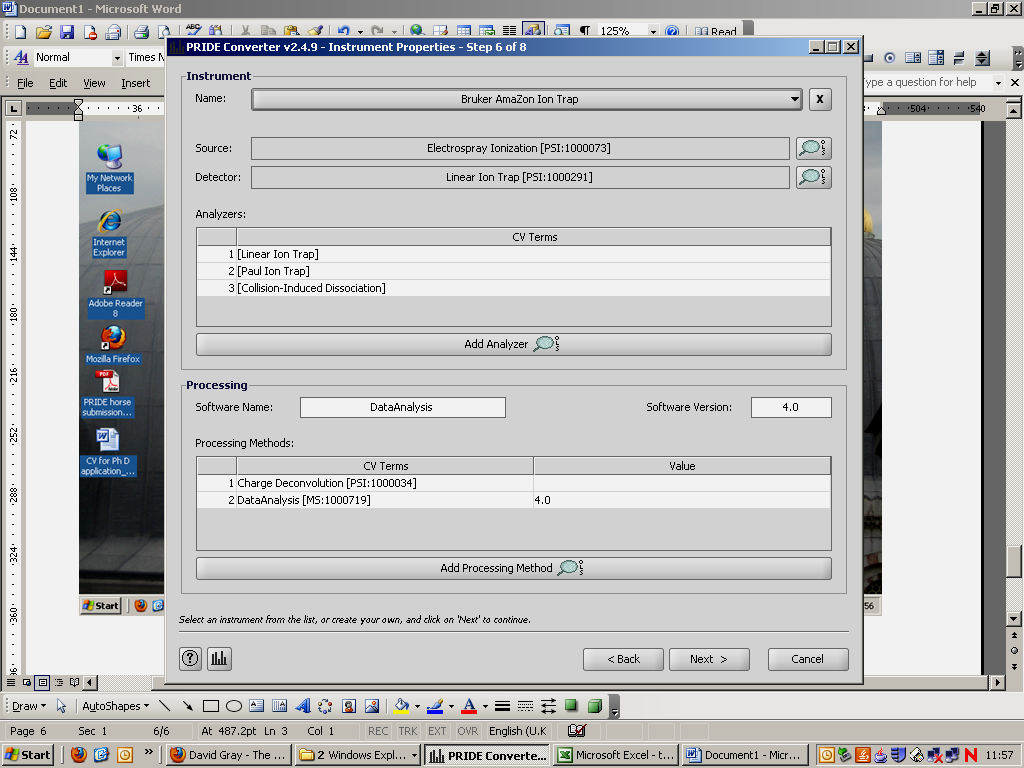


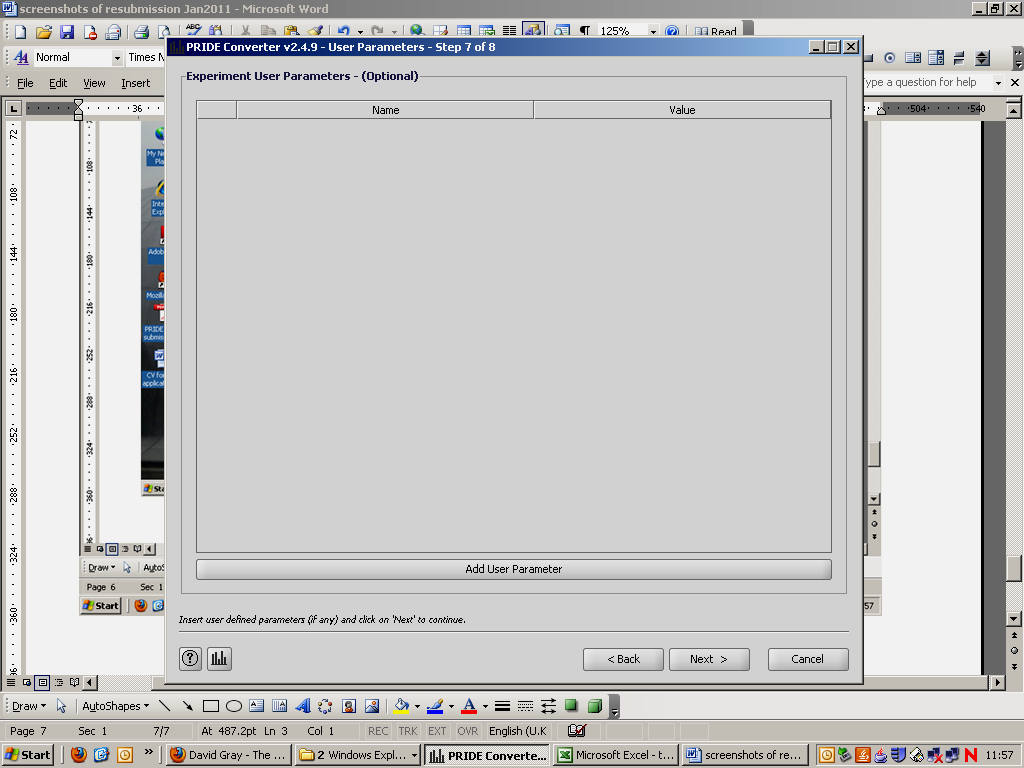


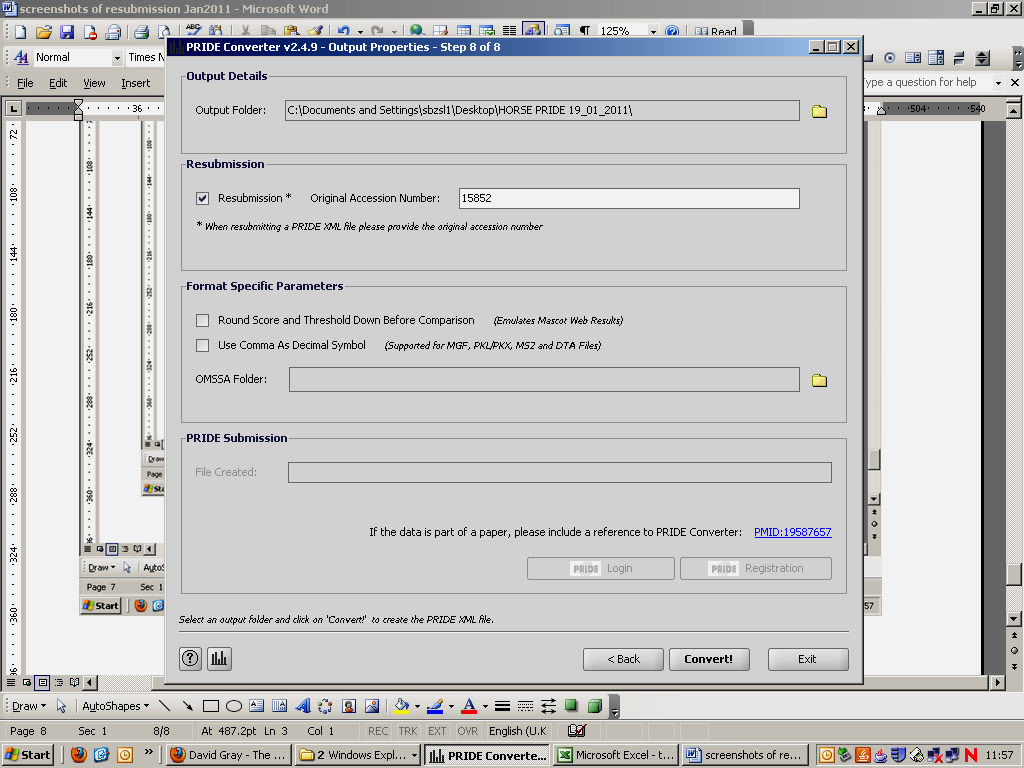


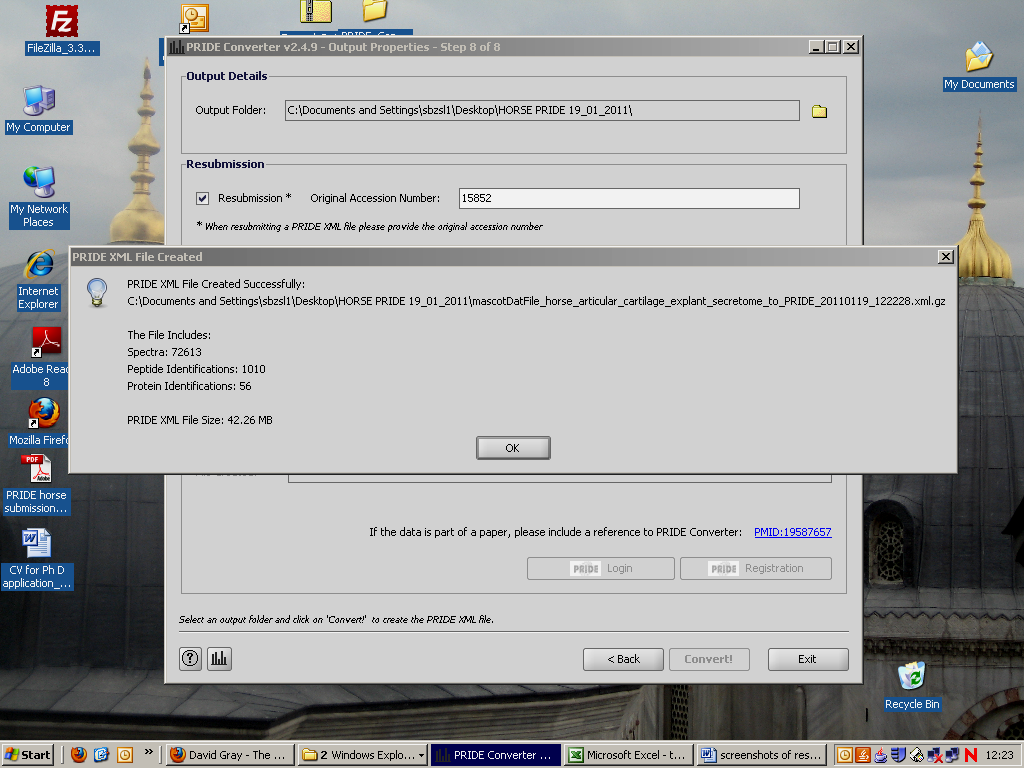


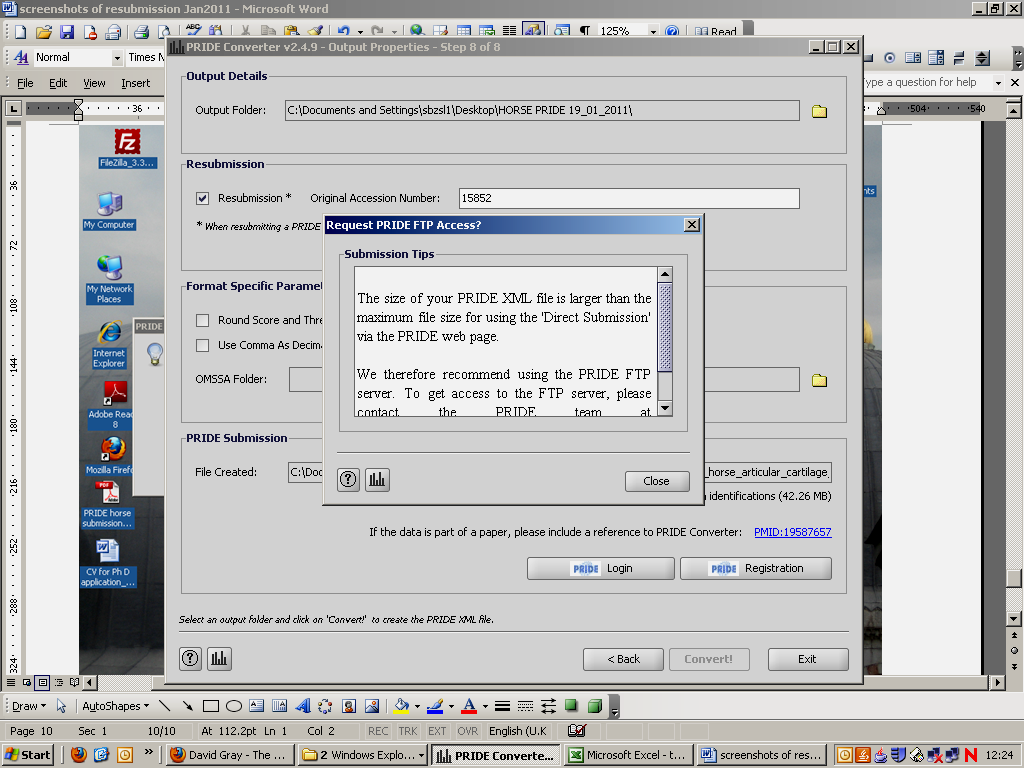


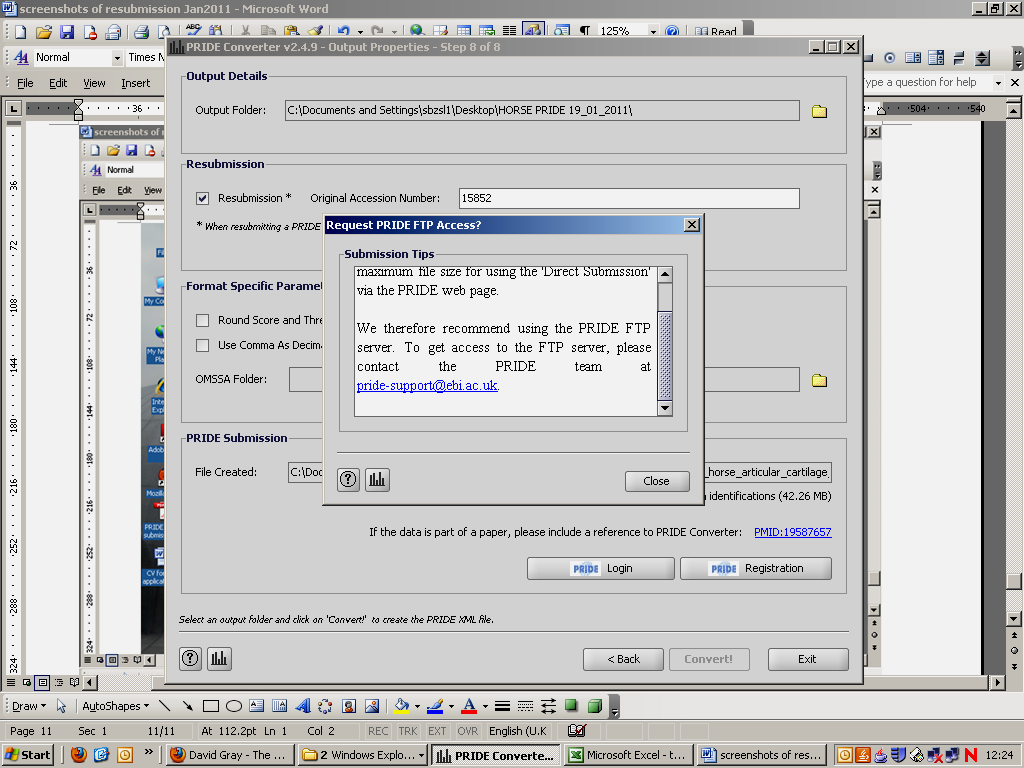


Submitted (or deleted) the wrong file via filezilla for the resubmission with identifications

therefore

Resubmitted the correct mascot.dat file


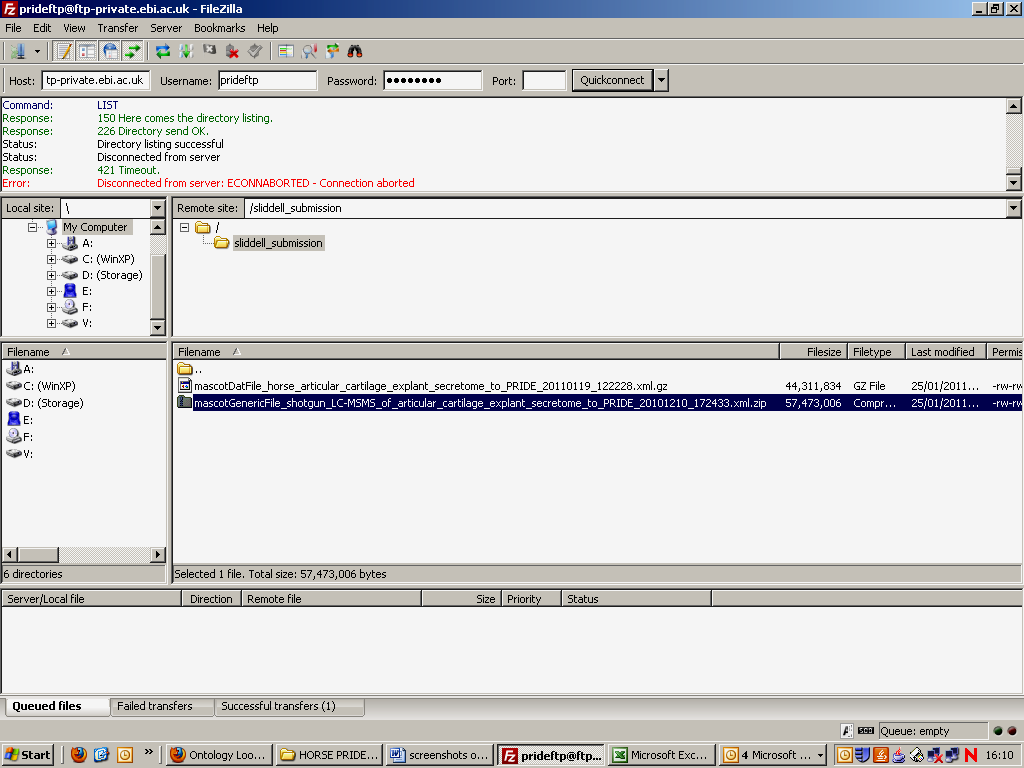

Supplement: Supplementary file 2 — Supplementary material 2. [file mmc2.doc]
