# Supplementary material for: High throughput proteomic analysis of the secretome in an explant model of articular cartilage inflammation
Source: J Proteomics. 2011 May 1;74(5-2):704–15. doi: 10.1016/j.jprot.2011.02.017 (PMC3078332; doi:10.1016/j.jprot.2011.02.017)
Supplement: Supplementary file 3 — How to reference PRIDE submissions. [file mmc3.doc]

## How to Reference PRIDE Submissons[¶](http://code.google.com/p/pride-converter/#How_to_Reference_PRIDE_Submissons)

Referencing PRIDE submissions in manuscripts:

- If still under some form of review:
  - The data is available in the PRIDE database [ref Martens et al 2005 (PMID: 16041671)] (www.ebi.ac.uk/pride) under accession numbers X and Y (username: reviewXYZ, password: ABC). The data was converted using PRIDE Converter [ref Barsnes et al 2009 (PMID: 19587657)] (http://code.google.com/p/pride-converter).
- If at galley proof stage:
  - The data is available in the PRIDE database [ref Martens et al 2005 (PMID: 16041671)] (www.ebi.ac.uk/pride) under accession numbers X and Y. The data was converted using PRIDE Converter [ref Barsnes et al 2009 (PMID: 19587657)] (http://code.google.com/p/pride-converter).

Ms ID pr-2010-00760R2 Second Revised Version:

***Submission of MS Data to PRIDE***

The MS data was submitted to the PRIDE repository (<http://www.ebi.ac.uk/pride/>) at the European Bioinformatics Institute under the accession numbers X and Y (username: reviewXYZ, password: ABC). The open source bioinformatic tool PRIDE Converter was used to facilitate uploading the data into PRIDE [32].

**After acceptance for publication, before or at page proof stage, this should become**

The MS data was submitted to the PRIDE repository (<http://www.ebi.ac.uk/pride/>) at the European Bioinformatics Institute under the accession numbers X and Y. The open source bioinformatic tool PRIDE Converter was used to facilitate uploading the data into PRIDE [32].
